# Effects of Oxathiapiprolin on the Structure, Diversity and Function of Soil Fungal Community

**DOI:** 10.3390/toxics10090548

**Published:** 2022-09-19

**Authors:** Yuxuan Chen, Fengwen Zhang, Bin Huang, Jie Wang, Haixia Huang, Zhanfeng Song, Shiying Nong, Chongjun Huang, Jianyu Wei, Haijiang Jia

**Affiliations:** 1 Tobacco Research Institute, Chinese Academy of Agricultural Sciences, Qingdao 266101, China; 2 School of Plant Protectio, Shandong Agricultural University, Taian 271000, China; 3 Tobacco Research Institute of Baise, Baise 533000, China; 4 Raw Material Technology Center of Guangxi Tobacco, Nanning 530001, China

**Keywords:** oxathiapiprolin, microbial community, diversity, functional prediction

## Abstract

Pesticides can affect non-target microorganisms in the soil and are directly related to soil microecological health and environmental safety. Oxathiapiprolin is a piperidinyl thiazole isoxazoline fungicide that shows excellent control effect against oomycete fungal diseases, including late blight, downy mildew, root rot, stem rot, and blight. Though it can exist stably in the soil for a long time, its effects on soil microbial structure and diversity are not well investigated. In the present study, the effects of oxathiapiprolin on the abundance and diversity of soil fungal communities in typical farmland were studied. The results show that the abundance and diversity of soil fungi were increased by oxathiapiprolin treatment with differences not significant on the 30th day. Oxathiapiprolin was found to change the structure of soil fungal communities, among which Ascomycota and Mortierellomycota were the most affected. Undefined saprophytic fungi increased in the treatment groups, and the colonization of saprophytic fungi can act as a major contributor to the function of soil microbial communities. This study lays a solid foundation regarding environmental behavior with the use of oxathiapiprolin in soil and details its scientific and rational use.

## 1. Introduction

With the development of modern agriculture and as a chemical control method, pesticides play an irreplaceable role in agricultural production. However, in recent days, countries across the globe are paying more attention to the protection of the environment by considering the impact of toxic pesticides on the stability of the ecosystem. Due to the low utilization rate of pesticides and non-standard application methods of farmers, about 80% of pesticides are found scattered in the soil, water, and atmosphere, causing indelible harm to the ecosystem [1,2].

Oxathiapiprolin is a new piperidinyl thiazole isoxazoline fungicide created by the DuPont Company. It is an inhibitor of the oxidized sterol binding protein (OSBP). The action sites are novel and highly effective against plant diseases caused by oomycetes, especially potato late blight caused by *Phytophthora infestans* [3]. However, past studies have shown that only a small proportion of pesticides are active on target crops, while a larger proportion accumulates in soils, sediments, and freshwater ecosystems through surface runoff and osmosis [4]. Zhou et al. found that the half-life of oxathiapiprolin in soil was 115 days [4], and its different enantiomeric structures were not significantly degraded within 150 days. It can be speculated that oxathiapiprolin may accumulate in soil and affect soil microecology. Studies show that the effect of pesticides on soil microecology can be judged by their effect on microbial communities. Therefore, it is necessary to pay attention to the changes in soil microbial communities under the effects of pesticides. At present, to the best of our knowledge, there are no reports available on the interaction between oxathiapiprolin and the soil ecosystem.

Soil microorganisms are organisms with the highest species diversity in the soil ecosystem. They are an integral part of soil material and play a vital role in soil transformation. In other words, they impact soil formation, development, and fertility. Soil microorganisms also promote the migration and transformation of nutrients required by plants [5,6]. They participate in soil life activities as a whole community, and changes in the bioactivity and structure of the community can sensitively reflect changes in soil quality, health status, and the ecosystem [7]. In this regard, the diversity and richness of soil microbial communities can be used to determine whether soil microecology is contaminated by pesticides [5]. Zhang et al. (2019) found that the copy number of soil bacteria was reduced by the fumigant 1,3-dichloropropene, which also changed the diversity and relative abundance of soil bacterial communities [8]. Application of sulfochlor and dichlorosulfochlor was found to increase the abundance of saprophytic fungi in the soil, and an increase in the number of functional bacteria may be related to the degradation of pesticides, thereby affecting soil nitrification and denitrification [6]. Soil fungi promote the energy flow and material circulation of the terrestrial ecosystem and maintain its normal operation. However, in recent years, scientists are more inclined to study soil bacteria rather than soil fungi [9]. As a fungicide, the effects of oxathiapiprolin on the soil fungal community remain unclear.

Given this, we investigated the risk assessment of oxathiapiprolin on soil microecology in pepper root rot soil. This study aims to provide a reference value for the risk assessment of oxathiapiprolin on diseased soil in the future.

## 2. Materials and Methods

### 2.1. Soil Sample Collection

Soil samples were collected from typical farmland with flat topography and stable soil texture in Tai’an City of Shandong Province. The five-point method was adopted for sampling. Before collecting, rotten leaves, weeds, and topsoil (about 1 cm from the soil surface) were removed. Soil on the arable layer was then collected with a shovel after disinfecting it with alcohol. The depth of sampling was approximately 20 cm. After collection, plant residues, gravel, and other sundries were removed from the soil samples, which were dried at room temperature, screened by 2 mm, completely mixed, and placed in an environment at 4 °C for further use.

Effects of oxathiapiprolin on soil microorganisms were tested in a constant temperature incubator in the laboratory. Soil moisture content was adjusted to about 60% of maximum field water capacity, and the soil was pre-cultured in a constant temperature incubator at 25 ± 1 °C for two weeks. Approximately 50 g of pre-cultivated soil was placed on an electronic balance tray when poisoning, to which a certain concentration of oxathiapiprolin–acetone solution was added, mixed evenly, stirred for 5 min, and ventilated for 2 h to completely volatilize the solvent. The sample was adjusted to 60% of the maximum water capacity in the field, transferred to a brown wide-mouth bottle, and the total weight was recorded. The sample was then kept in an incubator at 25 °C for dark culture. Three repetitions were made for each concentration when poisoning, and each repeated treatment was divided into three brown vials (corresponding to three sampling cycles). During the culture process, the water content was supplemented every 2 days to the initially recorded total bottle weight to maintain constant water content. According to the pesticide information network [10], the recommended dosage of 10% oxathiapiprolin oil dispersion in the field is between 195 and 300 mL/ha. Based on this, the concentrations of oxathiapiprolin in the present study were set as 0.2, 1.0, and 20.0 mg/kg, respectively, along with a blank control to explore the effects of different concentrations of oxathiapiprolin on the structure and diversity of soil microbial communities. The degradation half-life of oxathiapiprolin in soil was previously reported as 7.6–12.0 days. Based on this, sampling time was determined on the 7th, 15th, and 30th days to measure both soil microbial community structure and diversity.

Surface soil was collected on the 7th, 15th, and 30th days, stored at −20 °C for extraction of soil DNA, and labeled LCK, L02, L1, and L20 (LCK, L02, L1, and L20 represent blank control, 0.2, 1, and 20 mg/kg, respectively).

### 2.2. DNA Extraction, Amplification, and High-Throughput Sequencing

A special kit for soil microbial metagenomic DNA extraction (FastDNA SPIN Kit for Soil, MP) was used. The ITS1 region of the fungal ITS gene was amplified by polymerase chain reaction (PCR) using primers ITS5-1737F (GGAAGTAAAAGTCGTAACAAGG) and ITS2-2043R (GCTGCGTTCTTCATCGATGC), respectively. The PCR reaction system (30 Μl) was made up of 15 μL Phusion Master Mix (2×), 1.5 μL (3 μM) forward primer, 1.5 μL (3 μM) reverse primer, 10 μL (10 ng) gDNA (1 ng/μL), and 2 μL ddH_2_O. PCR reaction steps were as follows: pre-denaturation at 98 °C for 1 min, followed by 30 cycles of deformation at 98 °C for 10 s, annealing at 50 °C for 30 s, elongation at 72 °C for 30 s, and a final extension at 72 °C for 5 min. Based on concentration, all PCR products were mixed in equal amounts. After quantification and quality control, the qualified PCR products were used to construct a DNA library. Paired-end 250 bp sequencing was performed using an Illumina HiSeq platform (Majorbio Bio-pharm Technology Co., Ltd., Shanghai, China).

### 2.3. Data Statistics and Analysis

Sequencing data were analyzed by the QIIME2 (v.2020.2) software on the Majorbio Cloud platform [11], and amplicon sequence variants (ASVs) were merged and clustered under the condition that the similarity was 100%. The Ribosomal Database Project (RDP) classifier based on the Bayesian algorithm was used for the taxonomic analysis of ASV representative sequences with a confidence of 0.7. The Bayesian method was used to classify and annotate ASVs. Mothur (v.1.30.0, Michigan State University, Michigan, USA) software was used to analyze the alpha diversity of fungal communities by Ace, Chao, Shannon, and Simpson indices [12]. Ace and Chao indices are used to characterize microbial community richness, while Shannon and Simpson indices are used for microbial community diversity. Redundancy analysis (RDA) was carried out by the R (v.3.3.1, The University of Auckland, Auckland, New Zealand) package ‘Vegan’ [13] and principal co-ordinate analysis (PCoA) by direct mapping based on Euclidean distance. Python (v.2.7.0, Python Software Foundation, Netherlands) software was used to analyze the relative abundance of species. Through linear discriminant analysis effect size (LEfSe), the samples were linearly discriminated according to different classification methods. Using linear discriminant analysis (LDA), species with significant differences were found in the classification of samples. Fungi Functional Guild (FUNGuild) was used as the classification tool and the microecological guild as the classification method [14]. Fungal communities were classified as species that could use resources in similar ways, and fungal functions were predicted and analyzed by the software FUNGuild (v.1.0, University of Minnesota, St. Paul, MN, USA). Data were initially processed with Microsoft Excel (v2010, Microsoft, Albuquerque, New Mexico, USA), and the differences in soil physicochemical factors and fungal diversity were compared by one-way analysis of variance (ANOVA) with the SPSS (v.23.0, International Business Machines Corporation, New York, NY, USA) software. Duncan’s multiple range test (DMRT) was used for multiple comparison tests between the groups.

## 3. Results and Discussion

### 3.1. Gene Sequence Data of Soil Samples

**Figure 1:**
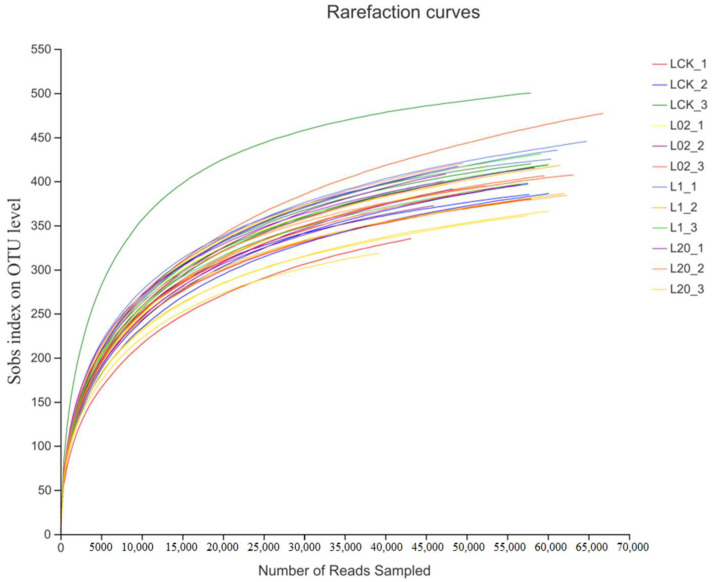
Sparsity curves of all samples. L indicates soil; CK represents no treatment; 02, 1, and 20 represent 0.2, 1.0, and 20.0 mg/kg, respectively.

A total of 2,027,706 optimized sequences were obtained by high-throughput sequencing of 36 soil samples (denoted by L), with an average length of 242.18 bp observed in the samples. Samples showed a minimum of 39,315 sequences and a maximum of 68,193 sequences. The reasonability of this sequencing can be judged by the dilution curve, as shown in Figure 1. Although the dilution curve did not reach a stable period towards the end, the curve became flat when the sequencing depth reached 50,000, thus indicating that a further increase in sequence number would not lead to the emergence of more new operational taxonomic units (OTUs). The confidence of the fungal community was considered high and could reflect the real situation of the fungal community and the structure of the rhizosphere soil samples comprehensively. This showed certain research significance and laid the foundation for further analyses. Thus, sequence depth and the number of data measured in this study were found reasonable.

### 3.2. Response of Alpha Diversity in Soil Fungi to Oxathiapiprolin

Diversity of soil fungal communities was quantitatively studied by alpha diversity analysis. Ace and Chao indices reflect species richness, while the Shannon index reflects the uncertainty of community diversity, which is diversity. The Simpson index indicates the evenness of community distribution. Table 1 shows the changes in the exposure of oxathiapiprolin to soil fungal diversity indices. Compared to the control group, the Ace index was significantly decreased on the 15th day (*p* < 0.05), with no significant changes on the 7th and 30th days (*p* > 0.05). In addition, during the whole sampling period, the Chao index increased in the treatment group with a high concentration on the 7th day (*p* < 0.05). However, no significant change in the Chao index was observed on the 15th and 30th days (*p* > 0.05), respectively. All concentrations of the Shannon index did not change significantly during the three sampling times (*p* > 0.05). It is worth mentioning that the Simpson index did not change significantly on the 7th and 15th days (*p* > 0.05), but did so on the 30th day, when it decreased to different degrees, especially in the 1.0 mg/kg treatment (*p* < 0.05).

These results indicate that the abundance and diversity of soil fungi can be increased by oxathiapiprolin treatment. On the 30th day, the number, abundance, and species diversity of fungi were not significantly different from those in the control group (*p* > 0.05), which could be due to a decrease in the residual amount of oxathiapiprolin in the soil with the change in time. Some studies have also shown that single or multiple fungicide applications over a certain period had differing effects on the abundance and diversity of soil fungi [15,16].

### 3.3. Response of Fungal Community Structure to Oxathiapiprolin

**Figure 2:**
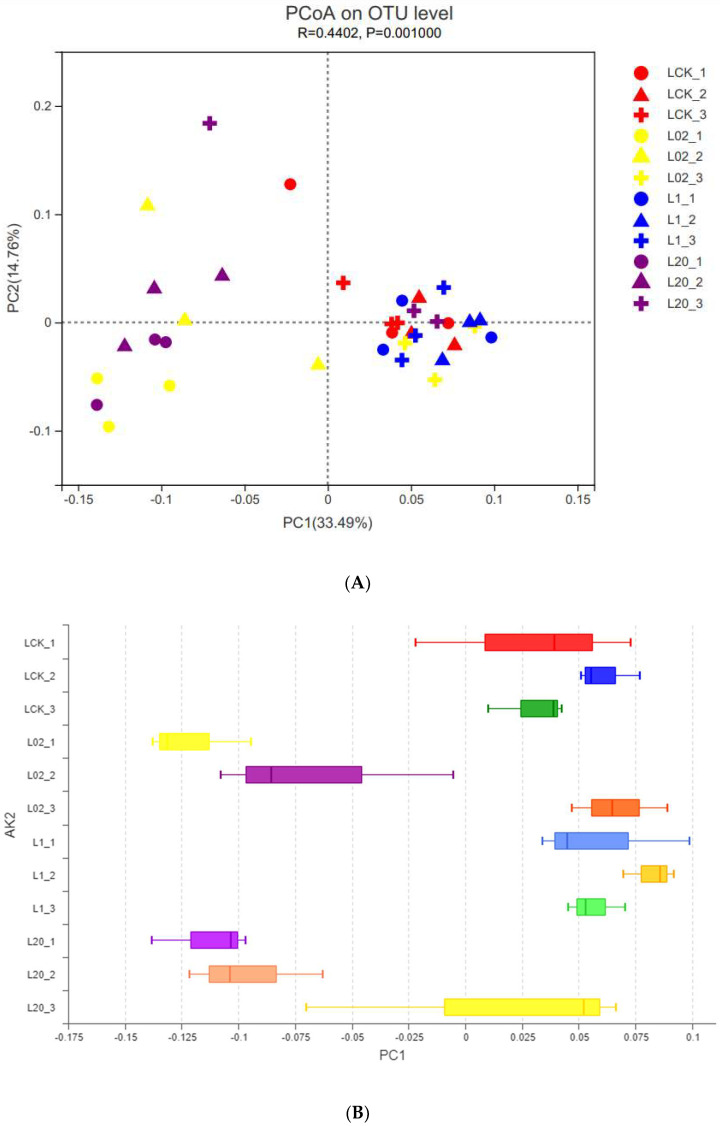
PCoA of fungal OTU community of soil samples with different treatments. (**A**) PCoA diagram depicts the Bray−Curtis heterogeneous matrix of the fungal community. (**B**) PC1 axis of PCoA in soil. L indicates soil; CK represents no treatment; 02, 1, and 20 represent 0.2, 1.0, and 20.0 mg/kg, respectively.

The effect of oxathiapiprolin on the phylum and genus populations was studied by PCoA at the OTU level (Figure 2). Interpretation degrees of the first two axes of PCoA were 33.49% and 14.76%, respectively. In Figure 2, different colors represent the different treatment groups, and the different shapes represent different treatment times. LCK was observed closest to L1, but far from L02 and L20. This indicated that both low and high concentrations of oxathiapiprolin had a great effect on soil fungi, while middle and low concentrations had little effect on soil fungi. At the same time, based on the shape, it was observed that the cross shape was more concentrated, while the circle and triangle shapes were scattered at different sampling times, indicating that with the extension of sampling time, the influence of drug concentration on soil fungi became weaker. By extending the PC1 (Project Cover 1) axis, different concentrations of oxathiapiprolin were isolated (Figure 2B), which indicated that the concentration of oxathiapiprolin was a key factor in changing the structure of the soil fungal community. In addition, observation of high-concentration treatment groups (1.0 and 20.0 mg/kg, Figure 2B) revealed that the sampling time could be separated in multivariate space, indicating that soil fungi in different treatments were also affected by sampling time.

**Figure 3:**
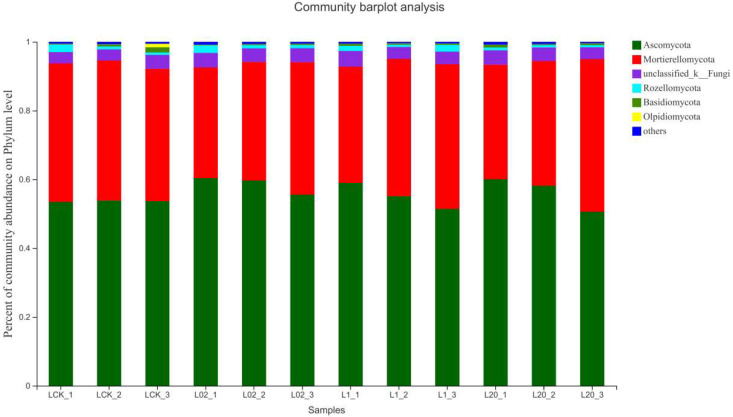
Composition of fungi in different soil communities. Relative abundance of different phyla in different communities. L indicates soil; CK represents no treatment; 02, 1, and 20 represent 0.2, 1.0, and 20.0 mg/kg, respectively.

**Figure 4:**
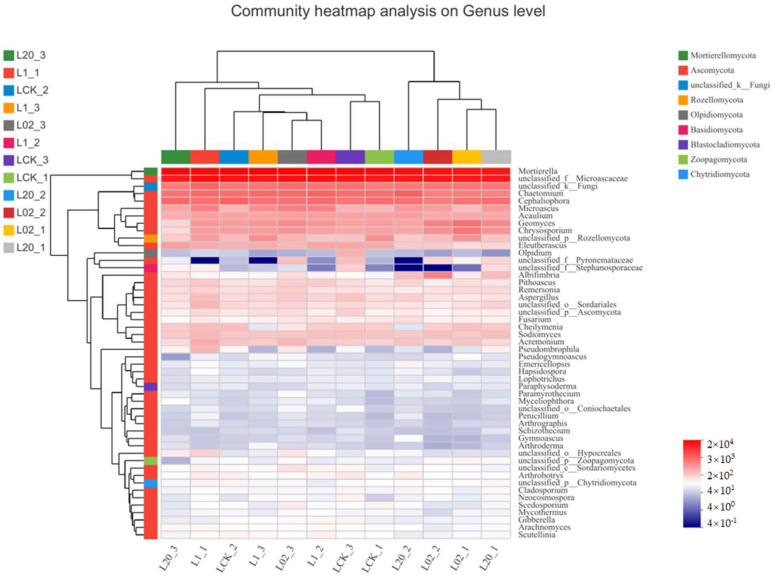
Heat map combined with cluster analysis of genus abundance composition of the soil (L) samples treated with oxathiapiprolin. A total of 30 genera are displayed. L indicates soil; CK represents no treatment; 02, 1, and 20 represent 0.2, 1.0, and 20.0 mg/kg, respectively.

Figure 3 and Figure 4 demonstrate the effect of the exposure of oxathiapiprolin on the relative abundance of phyla in soil. Ascomycota was found the most abundant and Mortierellomycota as the second most abundant, both accounting for more than 90% of the reads in the sample. When considering Ascomycota alone, an increase in all treatments was observed, except the 20 mg/L treatment group on the 30th day. On the contrary, the abundance of Mortierellomycota increased only at 20 mg/L on the 30th day and decreased in all other treatments. It is clear from the study that Ascomycota, a major component of soil saprophytic fungi, is also one of the decomposers of plant and animal bodies and feces in the soil [16]. The results show that the number of ascomycetes decreased in the middle and late stages of treatment, indicating that exposure to oxathiapiprolin can inhibit the colonization of certain fungi in this phylum. In addition, the abundance of Mortierellomycota showed an upward trend under oxathiapiprolin treatment, including a positive role in soil with low nutrient levels [16]. This shows that oxathiapiprolin can improve the abundance of beneficial bacteria in the soil. Cai et al. [17] found that the number of bacteria, actinomycetes, and fungi in the rhizosphere of diseased pepper plants was significantly higher compared to healthy pepper plants. Therefore, we hypothesized that the changes in the abundance of these two fungal genera may be involved in the occurrence, development, and spread of plant diseases, and oxathiapiprolin can be used to reduce their incidence.

**Figure 5:**
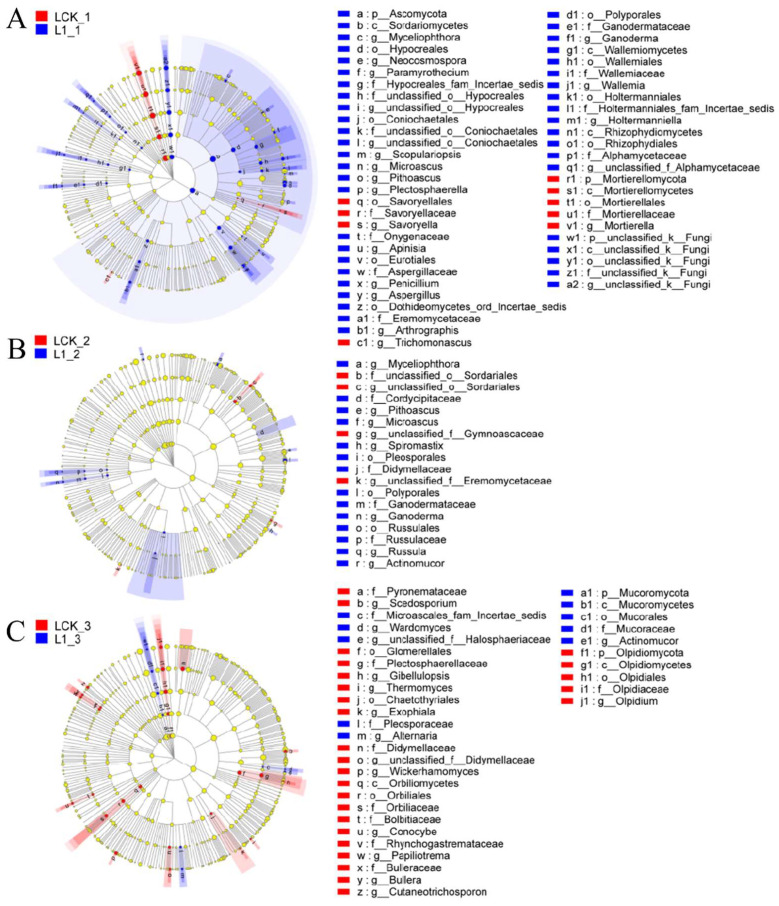
Phylogenetic distribution of fungal lineages in soil (L) treated with oxathiapiprolin. (**A**) 7 days after treatment; (**B**) 15 days after treatment; (**C**) 30 days after treatment. L indicates soil; CK represents no treatment; 02, 1, and 20 represent 0.2, 1.0, and 20.0 mg/kg, respectively. Circles indicate the level of phylogeny from domain to genus, and the diameter of each circle is proportional to the abundance of the group.

With the recommended dose of oxathiapiprolin in the field as 1 mg/kg, LEfSe multistage discriminant analysis was performed between oxathiapiprolin at a concentration of 1 mg/kg and the control group. As shown in Figure 5A, on the seventh day of treatment, Ascomycota was found significantly enriched by L1 treatment, while Mortierellomycota was significantly enriched by LCK. In addition, Hypocreales, Coniochaetales, Eurotiales, Polyporales, Wallemiales, Holtermanniales, Rhizophydiales, and a large number of undefined fungi were significantly enriched by L1 treatment, while Mortierellomycetes and Sovoryellales were significantly enriched by LCK. In terms of genus distribution, *Aspergillus*, *Penicillium*, *Pithoascus,* and *Microascus* were significantly enriched by L1 treatment, and *Mortierella*, *Trichomonascus,* and *Savoryella* were enriched by LCK. As shown in Figure 5B, Pleosporales, Russulales, and Polyporales were significantly enriched by L1 on the 15th day of treatment, while Sordariales was significantly enriched by LCK. In addition, *Microascus*, *Pithoascus*, *Myceliophthora*, *Spiromastix*, *Russula*, *Ganoderma*, and *Actinomucor* were significantly enriched by L1 treatment. Only three undefined genera were significantly enriched by LCK. As shown in Figure 5C, Olpidiomycota was significantly enriched by LCK on the 30th day of treatment. Glomerellales, Chaetothyriales, Orbiliales, and Olpidiales were significantly enriched by LCK, but only Mucorales was significantly enriched by L1. *Gibellulopsis*, *Thermomyces*, *Exophiala*, *Wickerhamomyces*, *Conocybe*, *Papiliotrema*, *Bullera*, *Cutaneotrichosporon*, *Olpidium,* and *Scedosporium* were significantly enriched by LCK. *Actinomucor*, *Wardomyces,* and *Alternaria* were significantly enriched by L1 treatment.

On the seventh day of L1 treatment, Ascomycota was found enriched with the regulation of soil nutrient cycling. As the largest phylum of fungi, Ascomycota degrades persistent organic matter, such as lignin and keratin. In addition, many ascomycetes could form symbiotic mycorrhiza and lichen, thus playing an important role in soil nutrient cycling [18]. On the 7th and 15th days of L1 treatment, Polyporales and *Microascus* were found enriched. It was reported earlier that the leavening of *Polyporus picipes* has inhibitory effects on seven common bacteria and six plant pathogens [19], which may inhibit the population of pathogens. *Microascus* was found closely related to the occurrence of human maxillary sinusitis, but its relationship with plant pathogens remains unclear to date [20]. The abundance of *Actinomucor* was significantly higher compared to LCK on the 15th and 30th days of L1 treatment. It was reported that the inhibition rate of 2- and 3-day-old buckwheat fermentation products of *Actinomucor elegans* on *Staphylococcus aureus* was as high as 100% [21], suggesting its certain antibacterial activity against plant pathogens. In addition, an increase in the abundance of *Alternaria* was observed after 30 days of L1 treatment, where *Alternaria* is also a potential biological resource. Certain species of *Alternaria* have high cellulase production, and some, such as endophytic bacteria, can produce vinblastine, paclitaxel, and other antibacterial drugs, which may be developed for biological control.

The results showed that both the concentration of oxathiapiprolin and sampling time after treatment can alter the fungal community in soil. Two-way ANOVA also demonstrated that the fungal community structure in the soils of both the treatment group and the control group was affected by oxathiapiprolin concentration and action time (*p* < 0.05). Du et al. (2018) used PCoA to find treatments composed of different doses of fungicides that significantly affect soil bacterial community structure, but not soil fungi [22]. However, studies have found that for triazol, a kind of foliar fungicide, soil fungal communities were more affected than bacterial communities [23]. Soil fungi play an important role in the carbon cycle, nitrogen cycle, and organic matter cycle to maintain soil ecological balance. However, this ecological balance is disturbed with the use of fungicides, as they kill some of the beneficial soil fungi. Studies on the effects of pesticides on soil microecology mainly focus on soil bacteria [24], with less emphasis on soil fungi. This is a somewhat astonishing as fungi are more closely related to soil microecology due to their role as decompressors in the ecosystem, as important plant pathogens, and their coexistence with plants as mycorrhizal fungi, all of which can serve as indicators of soil microecology. Therefore, studying the effects of pesticides on the diversity of soil fungal community is of great significance to improving plant and soil health, and deserves further attention.

### 3.4. Functional Prediction Analysis of Functional Changes in Soil Fungal Community after Oxathiapiprolin Treatment

**Figure 6:**
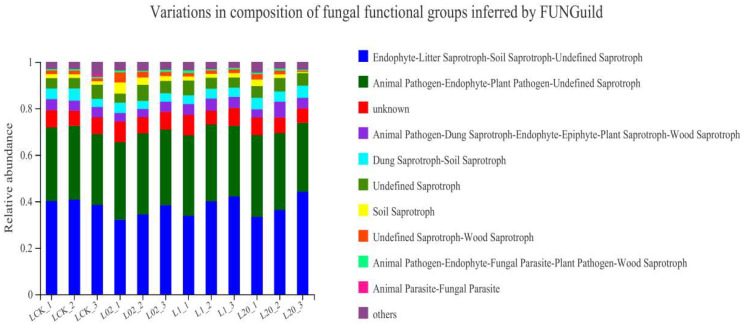
Fungal functional groups of soil inferred by FUNGuild. L indicates soil; CK represents no treatment; 02, 1, and 20 represent 0.2, 1.0, and 20.0 mg/kg, respectively.

Figure 6 shows the functional prediction diagram of soil fungi in Tai’an. Endophyte–Litter Saprotroph–Soil Saprotroph–Undefined Saprotroph, Animal Pathogen–Endophyte–Plant Pathogen–Undefined Saprotroph, and unknown were identified as the main functional members of the fungal community, with a majority (50%) being saprophytes. In all sampling sites, the abundance of undefined saprophytes increased after treatment with oxathiapiprolin, but still decreased compared to the blank control. At the later stage of sampling, there was no significant difference in the abundance of soil fungi between the treatment and the control groups, which may be due to the reduced influence of late agents in the soil and the restoration of soil fungi to their original state. On the whole, the main functional flora in the soil under study was saprophytic fungi, Ascomycota. The number of undefined saprophytic fungi in each treatment group increased after treatment with oxathiapiprolin. Colonization of saprophytic fungi can enhance the energy flow and material circulation of soil microecology, which is beneficial to plant growth and development [22]. At the same time, the abundance and diversity of soil fungi increased and soil stability improved with oxathiapiprolin treatment. Soil microbial diversity and abundance reflect the health and stability of an ecosystem [8]. Exposure to oxathiapiprolin did not show long-term effects on soil fungal communities. In this regard, we conclude that oxathiapiprolin has beneficial effects on soil ecology, and though it plays an important role in the process of plant resistance to various pathogens, its application does not increase soil ecological risks.

## 4. Conclusions

To study the possible effects of oxathiapiprolin on soil ecology, we evaluated the impact of oxathiapiprolin on soil fungi abundance, community structure, and function, and the following were concluded:

(1) Oxathiapiprolin can change the alpha diversity of soil fungi to different degrees, as shown by the increases in Ace, Chao, Shannon, and Simpson indices.

(2) Oxathiapiprolin can change the number and composition of soil fungi.

(3) According to FUNGuild functional prediction, the relative abundance of “undefined saprophytes” increased after treatment with oxathiapiprolin, which could be explained by the improvement of plant growth and quality after treatment with oxathiapiprolin.

Though this study established that oxathiapiprolin could affect soil microecology to a certain extent, especially the structure and function of soil fungal community, its long-term effects on soil fungi were within the permissible limits. In addition, the results of the indoor experiment are of great significance for us to understand the impact of oxathiapiprolin on soil fungal microecology. In the future, we will conduct outdoor experiments to verify this conclusion.

## Figures and Tables

**Table 1:** Soil fungal community diversity indices. L indicates soil; CK represents no treatment; 02, 1, and 20 represent 0.2, 1.0, and 20.0 mg/kg, respectively. Means (N = 3) within the same time period accompanied by the same letter are not statistically different (P = 0.05) according to Duncan’s new Multiple-Range test.

Diversity Index	Treatment	7 d	15 d	30 d
Ace	LCK	433.91 ± 14.16 a^1^	491.49 ± 14.84 a	436.03 ± 17.68 a
	L0.2	439.18 ± 12.30 a	458.35 ± 4.72 ab	447.94 ± 32.19 a
	L1	475.74 ± 5.46 a	441.50 ± 14.21 b	469.39 ± 21.93 a
	L20	501.48 ± 28.24 a	470.53 ± 0.58 ab	460.67 ± 16.24 a
Chao	LCK	433.68 ± 10.85 b	497.44 ± 19.33 a	434.35 ± 16.71 a
	L0.2	443.81 ± 7.26 b	463.60 ± 9.93 a	451.31 ± 7.26 a
	L1	482.98 ± 5.73 ab	446.22 ± 14.59 a	472.25 ± 5.73 a
	L20	505.79 ± 19.31 a	473.98 ± 2.26 a	471.26 ± 15.30 a
Shannon	LCK	3.03 ± 0.0116 a	3.2922 ± 0.0776 a	3.0472 ± 0.0126 a
	L0.2	3.02 ± 0.0355 a	3.2088 ± 0.0640 a	3.0195 ± 0.0440 a
	L1	3.20 ± 0.0485 a	3.0356 ± 0.1235 a	3.1958 ± 0.0445 a
	L20	3.09 ± 0.0601 a	3.1376 ± 0.0646 a	3.086 ± 0.0579 a
Simpson	LCK	0.1291 ± 0.0057 a	0.1095 ± 0.0069 a	0.1257 ± 0.0037 a
	L0.2	0.1206 ± 0.0057 a	0.1096 ± 0.0095 a	0.132 ± 0.0044 a
	L1	0.1196 ± 0.0091 a	0.1254 ± 0.0149 a	0.1083 ± 0.0040 b
	L20	0.1176 ± 0.0054 a	0.1146 ± 0.0072 a	0.1231 ± 0.0043 ab

^1^ The letters a, b and ab are significant markers, and different letters (a, b) between treatments indicate significant differences.

## References

[1] Yao X., Qiao Z., Zhang F., Liu X., Du Q., Zhang J., Li X., Jiang X. (2020). Effects of a novel fungicide benzovindiflupyr in Eisenia fetida: Evaluation through different levels of biological organization—ScienceDirect. Environ. Pollut..

[2] Tang F.H., Lenzen M., McBratney A., Maggi F. (2021). Risk of pesticide pollution at the global scale. J. Nat. Geosci..

[3] Pasteris R.J., Hanagan M.A., Bisaha J.J., Finkelstein B.L., Hoffman L.E., Gregory V., Andreassi J.L., Sweigard J.A., Klyashchitsky B.A., Henry Y.T. (2016). Discovery of oxathiapiprolin, a new oomycete fungicide that targets an oxysterol binding protein. Bioorg. Med. Chem..

[4] Pan X., Wu X., Liu N., Xu J., Liu X., Wu X., Feng Y., Li R., Dong F., Zheng Y. (2020). A systematic evaluation of zoxamide at enantiomeric level—ScienceDirect. Sci. Total Environ..

[5] Zhao H.-P., Wu Q.-S., Wang L., Zhao X.-T., Gao H.-W. (2009). Degradation of phenanthrene by bacterial strain isolated from soil in oil refinery fields in Shanghai China. J. Hazard. Mater..

[6] Zhang Y., Zhang J., Shi B., Li B., Du Z., Wang J., Zhu L., Wang J. (2021). Effects of cloransulam-methyl and diclosulam on soil nitrogen and carbon cycle-related microorganisms. J. Hazard. Mater..

[7] Maurya S., Abraham J.S., Somasundaram S., Toteja R., Makhija S. (2020). Indicators for assessment of soil quality: A mini-review. Environ. Monit. Assess..

[8] Zhang D., Ji X., Meng Z., Qi W., Qiao K. (2019). Effects of fumigation with 1,3-dichloropropene on soil enzyme activities and microbial communities in continuous-cropping soil. Ecotoxicol. Environ. Saf..

[9] Breulmann M., Schulz E., Weißhuhn K., Buscot F. (2012). Impact of the plant community composition on labile soil organic carbon, soil microbial activity and community structure in semi-natural grassland ecosystems of different productivity. Plant Soil.

[10] DuPont PD20160340. 2016.2.26–2026.2.29. http://www.chinapesticide.org.cn/hysj/index.jhtml.

[11] Bokulich N.A., Subramanian S., Faith J.J., Gevers D., Gordon J.I., Knight R., Mills D.A., Caporaso J.G. (2013). Quality-filtering vastly improves diversity estimates from illumina amplicon sequencing. Nat. Methods.

[12] Schloss P.D., Westcott S.L., Ryabin T., Hall J.R., Hartmann M., Hollister E.B., Lesniewski R.A., Oakley B.B., Parks D.H., Robinson C.J. (2009). Introducing mothur: Open-source, platform-independent, community-supported software for describing and comparing microbial communities. Appl. Environ. Microbiol..

[13] Kolde R. (2015). Pheatmap: Pretty Heatmaps. R Package. Version 1.0.8. http://cran.r-project.org/web/packages/pheatmap/index.html.

[14] Douglas G.M., Maffei V.J., Zaneveld J., Yurgel S.N., Langille M. (2019). PICRUSt2: An Improved and Extensible Approach for Metagenome Inference.

[15] Wu X., Xu J., Dong F., Liu X., Zheng Y. (2014). Responses of soil microbial community to different concentration of fomesafen. J. Hazard. Mater..

[16] Ju C., Xu J., Wu X., Dong F., Liu X., Zheng Y. (2016). Effects of myclobutanil on soil microbial biomass, respiration, and soil nitrogen transformations. J. Environ. Pollut..

[17] Yan C. (2002). Resources and Application of Soil Actinomycetes in Qinghai Plateau (East). Master’s Thesis.

[18] Hansen K., Perry B.A., Dranginis A.W., Pfister D.H. (2013). A phylogeny of the highly diverse cup-fungus family Pyronemataceae (Pezizomycetes, Ascomycota) clarifies relationships and evolution of selected life history traits. Mol. Phylogenet. Evol..

[19] Dong Y., Zhang A., Ma H., Li X., Gao J. (2007). Preliminary study on antibacterial activity of fermentative metabolites of Porus fuscifolia. J. Northwest For. Coll..

[20] Aznar C., Bievre C.D., Guiguen C.J. (1989). Maxillary sinusitis from *Microascus cinereus* and *Aspergillus repens*. Mycopathologia.

[21] Tang T., Chen X., Song F., Wang F., Li L. (2017). Screening of polypeptide strains from Tartary buckwheat by solid state fermentation. Microbiol. Bull..

[22] Du P., Wu X., Xu J., Dong F., Liu X., Zheng Y.J. (2018). Effects of triuralin on the soil microbial community and functional groups involved in nitrogen cycling. J. Hazard. Mater..

[23] Santísima-Trinidad A., María M., Diéz-Rojo M., Pascual J.A., Ros M. (2018). Impact of foliar fungicides on target and non-target soil microbial communities in cucumber crops. Ecotoxicol. Environ. Saf..

[24] Hooper D.U., Chapin F.S., Ewel J.J., Hector A., Inchausti P., Lavorel S., Lawton J.H., Lodge D.M., Loreau M., Naeem S. (2005). Effects of biodiversity on ecosystem functioning: A consensus of current knowledge. Ecol. Monogr..

